# Telehealth for the Initial Evaluation of Musculoskeletal Disorders: Qualitative Study of Patients, Health Care Providers, and Key Stakeholders in the Province of Quebec in Canada

**DOI:** 10.2196/72901

**Published:** 2025-07-24

**Authors:** Raphaël Vincent, Pauline Lemersre, Geneviève Ferland, Annie Bélanger, Audrey-Anne Cormier, Kadija Perreault, Jean Sébastien Roy, Nicolas Pinsault, Dahlia Kairy, François Desmeules

**Affiliations:** 1 School of Rehabilitation, Faculty of Medicine, University of Montreal, 7077 Parc Avenue, Montreal, QC, H3N 1X7, Canada, 1 514 343 6791; 2 Maisonneuve-Rosemont Research Center Hospital, University of Montreal Affiliated Research Center, Montreal, QC, Canada; 3 Centre for Interdisciplinary Research in Rehabilitation of Greater Montreal, Montreal University Institute for Physical Disability Rehabilitation, Montreal, QC, Canada; 4 University of Sherbrooke, Sherbrooke, QC, Canada; 5 School of Rehabilitation Sciences, Faculty of Medicine, Laval University, Quebec City, QC, Canada; 6 Center for Interdisciplinary Research in Rehabilitation and Social Integration, Quebec City, QC, Canada; 7 IFPS (Health Professional Training Institute), Department of Physiotherapy, Grenoble Alpes University, Grenoble, France; 8 ThEMAS (Techniques for the Evaluation and Modeling of Health Actions) Team, TIMC (Medical Engineering and Complexity Techniques) Laboratory, UMR (Joint Research Unit) CNRS-UGA (The French National Centre for Scientific Research–Université Grenoble Alpes) 5525, Grenoble Alpes University, Saint-Martin-d'Heres, France

**Keywords:** diagnosis, musculoskeletal disorders, physical examination, qualitative study, remote evaluation, telehealth

## Abstract

**Background:**

Access to care for patients with musculoskeletal disorders (MSKDs) remains a significant challenge. Telehealth has emerged as a promising solution to improve access to care. However, conducting initial evaluations of MSKDs remotely raises concerns about patient safety and clinical efficacy due to the necessary adaptations required for a clinical examination and the challenges of obtaining an accurate and reliable diagnosis.

**Objective:**

We aim to explore the use of telehealth for the initial evaluation of MSKDs in the province of Quebec, Canada. Through semistructured interviews with selected patients, health care providers, and other key stakeholders involved in telehealth, this study aims to provide a comprehensive and detailed understanding of its application, benefits, and challenges.

**Methods:**

Semistructured interviews were conducted in the province of Quebec with patients, clinicians, telehealth software specialists, and professional bodies’ representatives. Five tailored interview guides were developed using the Consolidated Framework of Implementation Research and the Framework of Mathieu-Fritz and Esterle for the study of telehealth interventions. The themes explored included participants’ prior experiences with telehealth, perceived strengths and limitations of telehealth, particularly regarding the initial evaluation and diagnosis of new patients, and the current global environment of telehealth use. All interviews were transcribed verbatim, and a reflexive thematic analysis was performed using the Mathieu-Fritz Framework.

**Results:**

Thirty-eight participants, including patients (n=11), health care providers (family physicians and musculoskeletal medical specialists: n=11; and physiotherapy professionals: n=10), telehealth software specialists (n=2), and representatives from professional bodies (n=4), shared their perspectives on telehealth for the initial evaluation of MSKDs. Five key themes emerged: (1) several participants viewed telehealth, including remote evaluations, as a solution to improve access to care; (2) patients and health care providers reported that a remote evaluation was more appropriate for simpler MSKD presentations; (3) some health care providers expressed concerns about the potential for an increase in diagnostic errors and the challenges of performing all usual components of a standard MSKD physical examination remotely; (4) patients expressed doubts about their ability to effectively perform certain tasks or tests on themselves; and (5) broader challenges were also highlighted by all participants, such as the impact on the patient-clinician relationship, access to appropriate hardware, digital literacy, and confidentiality concerns.

**Conclusions:**

Telehealth is seen as a valuable solution to improve access to care for patients with MSKDs, especially for simpler cases or urgent needs. However, remote physical examination poses challenges associated with concerns about diagnostic accuracy and limited remote physical examination procedures and components. Effective implementation will likely require more evidence-based guidelines, provider training on remote techniques and strategies to maintain patient-provider relationships. Addressing access to technology, digital literacy, and privacy concerns is also essential to ensure equitable adoption and to optimize telehealth in musculoskeletal care.

## Introduction

### Background

Musculoskeletal disorders (MSKDs), which affect muscles, bones, and joints, encompass conditions such as neck or back pain, tendinopathies, joint sprains, or articular conditions [1,2]. With global aging and lifestyles becoming increasingly sedentary, MSKDs have grown into an important burden on health care systems worldwide [1,2]. Access to care is a major challenge for patients with MSKDs, and wait times are steadily increasing in several Western countries [3-5]. Early access to an initial consultation with a health care provider is associated with improved patient outcomes [5]. This consultation should include a comprehensive physical evaluation to ensure an accurate diagnosis, to exclude any red flags or serious urgent conditions in order to provide safe and efficient care [2,6]. This process typically includes a structured review of the patient’s medical history, specific MSKD-related pain, other symptoms, and related disability, followed by a physical examination that assesses posture, functional movements, specific mobility, strength, and incorporates orthopedic clinical tests. Additionally, this evaluation often includes screening for different factors associated with poorer prognoses such as psychological or work-related factors, ensuring that all the aspects of the condition are addressed [2].

Telehealth care models, using videoconferencing or phone calls, offer opportunities to improve access to care by reducing travel needs for patients with limited mobility, for those with specific needs, or better access for citizens in rural areas [7-11]. Telehealth has been shown to be a valid alternative to traditional in-person care for the remote management of patients with MSKDs following an initial in-person evaluation [12-16]. However, telehealth remains underused for the initial evaluation of patients with MSKDs, despite emerging evidence of its advantages reported in the literature [17,18]. Clinical measurements, such as pain or range of motion assessed remotely, have shown good validity and excellent reliability [17,19]. A few studies conclude that remote diagnoses have been valid for lower or upper limb MSKDs and, in some cases, for more acute MSKDs [17,18]. Limited evidence is available for spine or degenerative MSKDs, such as osteoarthritis, which are among the most common causes of musculoskeletal pain [1,17,18].

Despite growing evidence supporting remote care for MSKDs, including the initial patient evaluation, the lack of direct in-person contact between patients and providers raises concerns among patients, health care providers, and other stakeholders [20-25]. Remote interventions are often perceived as less effective, less safe, and associated with a weaker therapeutic relationship compared to traditional in-person care [20-25]. Patients and health care providers also highlight the need to develop new habits referred to as “webside manner” in the literature that accommodate the challenges associated with communication during remote consultations [26-28]. In addition to the challenges related to changes in clinical practice and patient-provider interactions, other issues were also highlighted, particularly those linked to the use of technology (such as digital literacy) and organizational aspects (such as insurance coverage of care) [20,22,24]. Mathieu-Fritz and Esterle [29] proposed a theoretical framework that connects all these various dimensions of a medical consultation impacted by the use of telehealth: clinical, social, technical, and organizational. These 4 dimensions are presented as key components to consider for analyzing telehealth and ensuring the success of remote consultation [29].

Perceptions on the initial remote evaluations of new patients with MSKDs are yet to be thoroughly explored [23,25]. Studies report that many providers are reluctant to perform remote initial evaluations and express a lack of confidence in remote diagnoses [23,25]. Currently, there is a lack of evidence on the perspectives of stakeholders, including patients, telehealth software specialists, and professional bodies, regarding the initial remote evaluation of patients with MSKDs. Furthermore, other possibilities offered by a first initial remote consultation, such as triage or prioritization, remain underexplored, highlighting the need to better understand how remote evaluations can be integrated into care pathways of patients with MSKDs.

### Objective

This qualitative study aims to explore the use of telehealth for the initial evaluation of MSKDs in the province of Quebec, Canada. Through semistructured interviews with selected patients, health care providers, and other key stakeholders involved in telehealth, this study aims to provide a comprehensive and detailed understanding of its application, benefits, and challenges.

## Methods

### Study Design

An exploratory qualitative study was conducted using semistructured interviews with various stakeholders in the province of Quebec, Canada, regarding the use of remote evaluations for diagnosing patients with MSKDs [30]. This publication conforms to the COREQ (Consolidated Criteria for Reporting Qualitative Research) checklist (Checklist 1) [31].

### Ethical Considerations

This study was approved by the Health Research Ethics Committee of the Centre intégré universitaire de santé et de services sociaux de l’Est-de-l’Île-de-Montréal (2024‐3396) in Montreal, Quebec, Canada. Written informed consent was obtained from all participants before the interviews. Participants were free to withdraw from this study at any time. All data transcripts used for analysis were deidentified. A CAD $50 (US $36.55) compensation was offered to patients participating in this study. No compensation was offered to the clinician or other stakeholder participants.

### Participants

Participants included patients with MSKDs, family physicians, musculoskeletal physician specialists, physiotherapists and physiotherapy technologists, telehealth software specialists, and representatives from professional bodies such as professional associations and regulatory colleges [32]. Physiotherapist technologists are rehabilitation professionals with a 3-year college training program. They work in collaboration with physiotherapists and physicians and may participate in patient assessment, always under the supervision of a physiotherapist or a physician. Eligible participants for this study were the following: (1) 18 years or older, (2) legally able to consent, and (3) able to understand or speak French.

Different sampling methods were used during the recruitment. Patients were recruited using purposive and opportunistic samplings, from a list of participants who consented to be recontacted from a previous study on management of MSKDs in emergency departments, or directly during a visit to the outpatient orthopedic clinic at the Maisonneuve-Rosemont Hospital [33]. Patients were recruited from 2 clinical settings to explore the perceptions of individuals receiving care in both primary and specialized care contexts. Health care providers were recruited using convenience and snowball sampling, through announcements made on social media professional groups and contacts of the research team. Particular attention was given during the recruitment of health care providers to ensure representation from various professions involved in the evaluation of MSKDs, and from diverse clinical settings. Telehealth software specialists and representatives from professional bodies were recruited using purposive sampling through direct solicitations. Written informed consent was obtained from all participants.

### Data Collection

Five interview guides (Multimedia Appendix 1) were created and adapted for each category of participants: (1) patients, (2) family physicians and medical musculoskeletal specialists, (3) physiotherapists and physiotherapy technologists, (4) professional bodies, and (5) telehealth software specialists. These guides were developed using 2 theoretical frameworks to enhance the credibility of our results. The Consolidated Framework of Implementation Research was used to identify factors that could impact the implementation of an intervention and the theoretical framework for telehealth consultation described by Mathieu-Fritz and Esterle [29] was used to identify dimensions of a medical consultation that could be impacted by the use of telehealth [34]. The interview guides were adapted to explore specific dimensions with each group: the personal, clinical, and social dimensions as well as patient-provider interactions for patients and provider participants, the organizational and regulatory dimensions with professional bodies, and the technology-related dimensions with telehealth specialists. A literature review also informed the guide development. The themes explored included experiences with telehealth (previous experiences, difficulties, and strategies to mitigate problems), the perceived strengths and limitations of telehealth for the initial evaluation, and the remote diagnosis of new patients with MSKDs. Sociodemographic information was collected at the end of the interview.

Individual semistructured interviews were conducted by a member of the research team (RV) via phone, videoconferencing (Zoom Inc), or in person, according to participants’ preferences. RV is a PhD candidate in rehabilitation sciences and a physiotherapist. RV had previous experience doing qualitative research and semistructured interviews. As a clinician, he also experienced telerehabilitation care with several patients. A single interview was conducted for each participant. Participants were recruited until enough informative power was reached [35]. Data sufficiency was determined by considering all factors and domains in the 2 theoretical frameworks and the literature review, and researchers noted that they were explored during the interviews with several participants [35,36]. Although data saturation was not a primary objective of this project, it was reached for both patients and health care providers, as no new themes emerged despite conducting interviews with participants from diverse backgrounds.

Interviews were audio-recorded, transcribed verbatim, and deidentified by 1 trained member of the research team (RV). Transcripts were not returned to participants for comment and correction.

### Data Analysis

A reflexive thematic analysis was performed using the Braun and Clarke approach [30,37]. The first 2 interviews from each category of participants were independently coded using NVivo14 (QSR International Pty Ltd) by pairs of researchers (RV and PL or RV and GF). All had previous experience in qualitative research, coding, and analysis. PL had previously conducted a study on telehealth implementation using the framework by Trupia et al [38]. Codes were then compared, and an initial coding tree was created. The remaining interviews were subsequently coded by a single researcher (RV), with any new themes generated being incorporated into the analysis.

To enhance the validity of the coding tree, one of the remaining interviews was double-coded by RV and PL for each participant category. Multiple meetings were held to discuss the coding structure and relationships between themes, and the final coding tree was reviewed and validated by all coders and the 2 principal researchers (FD and DK). The theoretical framework of Mathieu-Fritz and Esterle [29] was used to organize the different themes.

All citations included in this paper were translated from French to English and verified by a second team member to ensure an accurate translation.

## Results

### Overview

A total of 38 interviews were conducted and lasted from 7 up to 75 (mean duration 36, SD 18) minutes. These included 11 interviews with patients, 11 with physicians (comprising 5 family physicians and 6 medical specialists), and 10 with physiotherapy professionals (8 physiotherapists and 2 physiotherapy technologists). Additionally, 2 interviews were conducted with telehealth software specialists, 2 interviews with representatives from physiotherapy associations (the *Association Québécoise de la Physiothérapie* and the *Fédération des Cliniques de Physiothérapie du Québec*), 1 with a manager from a private rehabilitation clinic group, and 1 interview with a representative from the *Ordre Professionnel de la Physiothérapie du Québec* (licensing body). Characteristics of patients and health care providers are presented in Tables1  2.

**Figure 1.**
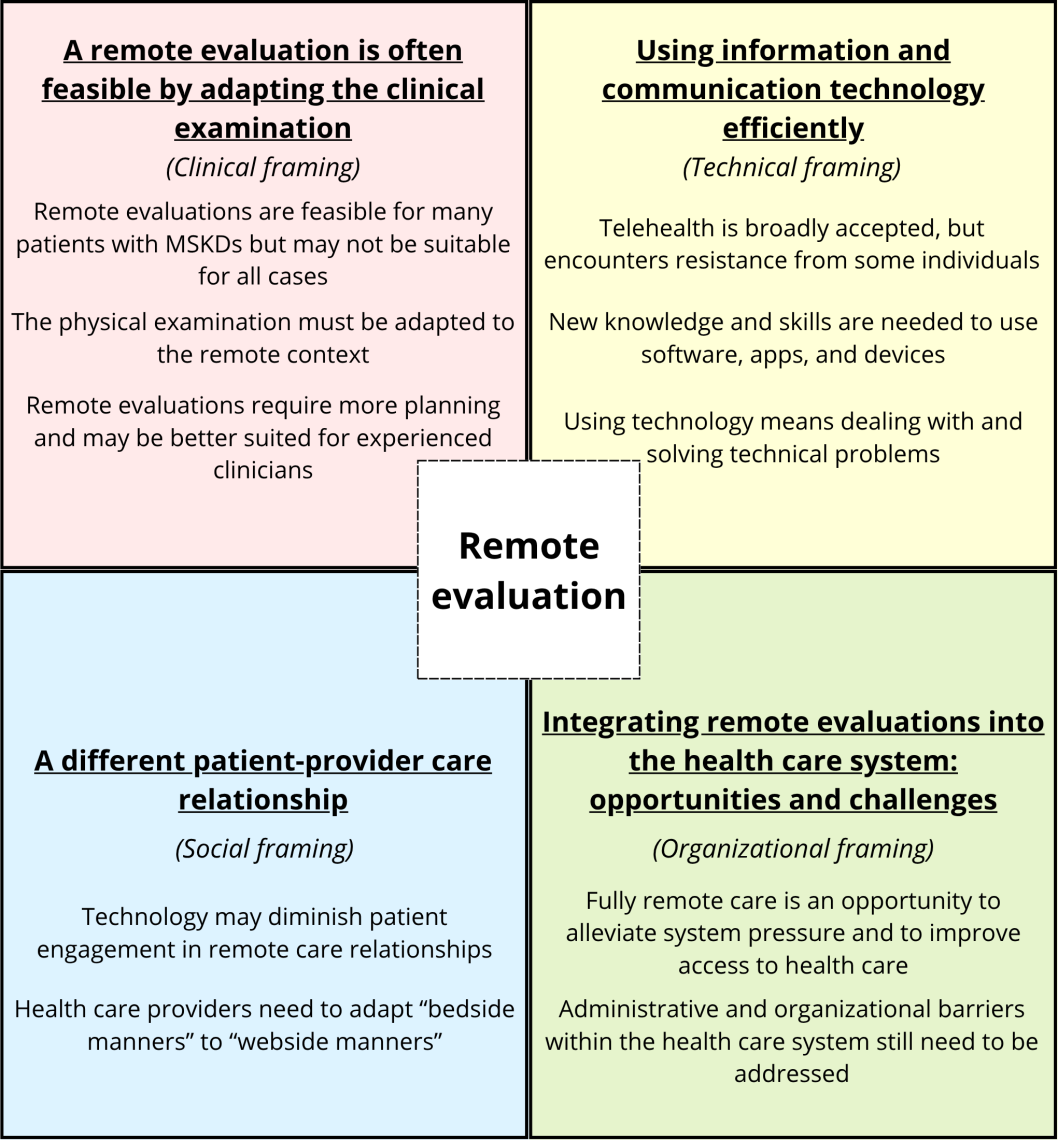
Main themes and subthemes. MSKD: musculoskeletal disorder.

Four major themes were identified related to the 4 dimensions described in the framework of Mathieu-Fritz and Esterle [29], but here in the context of an initial remote evaluation: (1) a remote evaluation is often feasible by adapting the clinical examination (clinical framing), (2) a different patient-provider care relationship (social framing), (3) using information and communication technology efficiently (technical framing), and (4) integrating remote evaluations into the health care system: opportunities and challenges (organizational framing). Main themes and subthemes are presented in Figure 1.

**Table 1.** Sociodemographic and practice-related characteristics of health care provider participants interviewed (n=21).

	Family physicians and medical specialists (n=11), n (%)	Physiotherapist (n=8) and physiotherapy technologists (n=2), n (%)
Gender		
Woman	4 (36)	5 (50)
Man	7 (64)	5 (50)
Clinical experience (years)		
<5	2 (18)	0 (0)
6‐10	6 (55)	4 (40)
>10	3 (27)	6 (60)
Primary work setting		
Family medicine group	4 (35)	1 (10)
Private physiotherapy practice	0 (0)	8 (80)
Orthopedic specialist clinic	4 (35)	0 (0)
Army medical service	0 (0)	1 (10)
Emergency department	1 (10)	0 (0)
Neurosurgery clinic	1 (10)	0 (0)
Physical medicine clinic	1 (10)	0 (0)
Urban area	9 (82)	6 (60)
Rural area	2 (18)	3 (30)
Practice setting sector		
Public	11 (100)	1 (10)
Private	0 (0)	8 (80)
Army medical service	0 (0)	1 (10)
Forms of telehealth used^a^		
Videoconferencing	3 (27)	10 (100)
Phone	10 (91)	2 (20)
Non	1 (10)	0 (0)

a Some health care providers used both phone and videoconferencing consultations.

**Table 2.** Sociodemographic characteristics of patient participants interviewed (n=11).

	Patients
Age (years), mean (SD)	58.0 (27-84)
Gender, n (%)	
Woman	6 (55)
Man	5 (45)
Highest education level completed, n (%)	
High school	3 (27)
College	3 (27)
University	5 (46)
Employment status, n (%)	
Employed	7 (64)
Retired	2 (18)
University student	1 (9)
Medical leave	1 (9)
Diagnoses (types of MSKDs)^a^, n (%)	
Peripheral disorders	8 (73)
Soft tissue injury	2 (25)
Osteoarthritis	2 (25)
Wrist inflammation	1 (12.5)
Rheumatoid arthritis	1 (12.5)
Ankle fracture	1 (12.5)
Patellar instability	1 (12.5)
Spinal disorders	3 (17)
Nonspecific low back pain	2 (67)
Neck pain with upper limb irradiation	1 (33)
Health care providers consulted^b^, n (%)	
Emergency physician	5 (45)
Orthopedic surgeon	7 (64)
Family physician	7 (64)
Physiotherapist	6 (55)
Rheumatologist	1 (9)
Previous telehealth experiences^c^, n (%)	
Phone	5 (45)
Videoconferencing	4 (36)
None	5 (45)

a MSKD: musculoskeletal disorder.

b No patient had experienced an initial remote evaluation for their musculoskeletal disorder.

c Some patients experienced both phone and videoconferencing consultations.

### Theme 1: Remote Evaluation Is Often Feasible by Adapting the Clinical Examination

Assessing a patient without physical contact and through a screen requires adapting the way health care providers conduct examinations; traditional hands-on assessments must be replaced or supplemented with visual observations, patient-reported information, and guided movements. This shift compels health care providers to adopt new strategies for gathering diagnostic insights effectively in a remote setting.

#### Remote Evaluations Are Feasible for Many Patients With MSKD but May Not Be Suitable for All Cases

A remote initial evaluation appears to be suitable for patients and health care providers when it applies to simple cases or presentations. For health care providers, remote evaluation can be used for common MSKDs, with typical clinical presentation, which are already easy to evaluate in person.

*I think that for a simple case where there is very little suspicion of a major structural lesion, with the history, with the subjective. I think there is no problem with a remote evaluation. It is a very good option*.

[Physiotherapist 2]

For some providers, more complex chronic MSKDs and screening for serious pathologies (presence of red flags) are also considered suitable for remote evaluation. They believe these evaluations can still be safe, precise, or valid, as they often rely heavily on the patient’s medical history and response to specific questions, rather than the physical examination.

On the other hand, medical specialists feel that remote evaluations are less suitable for their practice as they care for patients with more complex disorders. They emphasize the need for a precise diagnosis where physical examination plays a crucial role in decision-making and determining the need for surgery, for example.

*Often, the diagnoses I will have to make are a little more specific, a little more precise than just someone with a general complaint of shoulder pain, for example. In my clinical practice, the physical examination plays a big role. It is what helps me to identify surgical indications. It will also help me plan postoperative management*.

[Orthopedic Surgeon 1]

For patients, the suitability of remote evaluations is more closely linked to the stage of the MSKD, whether acute or chronic. They plan to use remote evaluation when a prompt assessment is needed, such as after a traumatic injury.

*I think that if I feel the need to consult quickly for my back problem, for example, I will be more inclined to accept a remote evaluation. If I have a fall, make a false move, something that happens suddenly. If I have had back pain for 10 years, I tell myself that it is not one more day or one less day that is going to change anything. And so I would be ready to wait longer to see a health care provider in-person.*

[Patient 1]

The emotional impact of the MSKD on their life was also perceived as an important factor. Most patient participants reported a preference for in-person evaluations if experiencing anxiety and stress about their condition. Loss of patient-provider physical contact was stated as an important reason for preferring an in-person meeting.

*I have the impression, but it is only an impression, that I would not have felt the same trust via videoconferencing as I did today when I saw him [the orthopedic surgeon], I sat next to him, I spoke to him, I shook his hand. It was very reassuring for me. And it [a remote consultation] lacks a bit of comfort for a problem that is important to us [...] With the emotions I am feeling today, I could not have done it by videoconferencing. I would not have had the same confidence that made me say to my doctor to operate on me as soon as he can*.

[Patient 10]

#### The Physical Examination Must Be Adapted to the Remote Context

Health care providers perceive that the subjective examination can be done remotely. Parts of the physical examination also appear to be achievable remotely without any adaptation, such as general or local observation and assessment of active range of motion. However, other parts of the physical examination, such as the evaluation of muscle strength or performing some special orthopedic tests, require adaptations.

*I think with video we can ask the patient to make movements, can you raise your arm? Can you bend forward? Can you bend over? Can you do a squat, you know? Clearly this would complete the interview, there may be a part of the physical examination that can be done remotely: joint range of motion, certain functional movements. Certain tests that typically require physical manipulation by a health care provider may be challenging. I have my doubts*.

[Family physician 3]

For certain MSKDs, such as neck or low back pain, the neurological evaluation presents an even greater challenge and may require further adaptation to ensure accuracy and reliability. Parts of the physical examination, such as palpation, appeared to be impossible to perform remotely for some providers, which leads to a loss of information.

*The muscular atrophy, I tell my residents all the time, it is palpable, it is not just visual, so you have to make the patient contract. Then you palpate, which means that no, it is not possible remotely*.

[Physiatrist 1]

The need to adapt certain parts of the physical examination and the inability to perform others remotely raises concerns among all the participants, including from representatives of professional organizations and even telehealth software specialists. Additionally, asking patients to perform some tests or maneuvers introduces challenges as providers question the patients’ lack of medical knowledge and abilities to perform these tasks. These questions about their abilities were also shared by the patients themselves.

*I do not trust myself to do the real test [...] I think I would introduce too much potential for error*.

[Patient 7]

#### Remote Evaluations Require More Planning and May Be Better Suited for Experienced Clinicians

Health care providers emphasize the importance of musculoskeletal care experience to properly manage patients with MSKDs remotely. Using telehealth is also perceived by providers, telehealth software specialists, and professional organizations as a distinct clinical practice requiring providers to rely “more on the brain and less on the hands” (physiotherapy technologist 2), emphasizing the need for practice and adaptation.

Providers report an increased workload during telehealth evaluations, even among those regularly using remote evaluations. Preparation for a remote evaluation extends beyond the evaluation itself and includes anticipating the need for specific items (for example: weights or dumbbells, a broom or stick, or a floor mat) and instructing patients on preparing the necessary materials and ensuring adequate visibility and space for the examination.

*I often told patients that if you have a laptop, it is ideal, you can put it down, you can play with the inclination of the screen easily, provide yourself with a little bench, you can use it to put your laptop on it. After two or three patients, you kind of know what works and what does not work so well, and then just explain it to the patient. Telling him what he needs to have, it is like preparing the home for the remote consultation, it helps to avoid these problems during the evaluation*.

[Physiotherapist 6]

### Theme 2: Different Patient-Provider Care Relationship

The initial evaluation, whether conducted in-person or remotely, is the first encounter between the patient and clinician. Both patients and providers reported anticipating a different type of interaction in the remote context compared to a typical in-person evaluation, which may impact the development of a strong therapeutic alliance.

#### Technology May Diminish Patient Engagement in Remote Care Relationships

A remote initial consultation may be less engaging for both patients and health care providers. Both parties highlight the challenge of creating a human connection, noting the limitations of not being physically present in the same room and the reliance on cameras and screens for communication. This virtual interface complicates the relationship by causing the loss of a part of the nonverbal cues and can contribute to a diminution of the feeling of a human relationship.

*My impression is that we lose a lot of the non-verbal in telehealth, which is often just as important as the verbal [...] And it is very, it is very impersonal, I think if it is just for the patient-physician relationship, it is a completely different experience*.

[Orthopedic Surgeon 5]

Since patients can stay home and no longer have to travel to a health care facility for the encounter, this may be seen as an advantage, usually, but some health care providers anticipate less commitment and engagement from patients.

*It is the patient who comes to the clinic. They engage in a process where they step out of their daily life to gain perspective on their life. But what if they are at home, in their kitchen, in their basement, in their office? I think the distance from their daily life is a little less, is a little less facilitated. And there is also the commitment of saying I take my car, I move, I am going to meet someone who is going to help me.*

[Physiotherapist 8]

#### Health Care Providers Need to Adapt “Bedside Manners” to “Webside Manners”

Beyond the challenges associated with the use of technology, the health care provider’s behavior is seen by patients as a key factor in the success of a consultation.

*I once had a bad in-person consultation with a doctor. When I entered the office, I greeted him, but he did not respond. He was looking at his file. So I said to him, “Hey, I greeted you.” It is important for me not to be treated like a number. I want to be treated like a human being*.

[Patient 9]

All patients and providers interviewed emphasized the importance of establishing “webside manners” to provide a satisfying and effective experience. Webside manners cover different aspects of care from the informed consent of patients to use telehealth to using new communication strategies. These strategies aim to enhance exchanges and to facilitate collaboration through the “virtual interface,” such as providers being more demonstrative or speaking louder, slower, and using simpler vocabulary.

*I had to adapt my vocabulary. […] I had to be very, very attentive with the patient, and I could not allow myself to take notes during the consultation. I also had to speak much louder. I was not used to any of this because it is not how I conduct my in-person consultations. I ended my days more tired, even if I had seen half as many patients.*

[Physiotherapist 5]

### Theme 3: Using Information and Communication Technology Efficiently

Patients and providers must rely on information and communication technology to conduct a remote evaluation. Some patients and health care providers express reluctance or refuse to use such technologies in health care.

#### Telehealth Is Broadly Accepted, but Encounters Resistance From Some Individuals

Most patients and health care providers agree to use technology such as videoconferencing in health care. However, some participants, particularly providers, strongly oppose remote care that they consider incompatible with their clinical practice preferences.

*For me, this is very personal. The pleasure I get is interacting with my patients. If I had wanted to do teleconsultation, I would have become an accountant, I would have been entering data into an Excel file all my life. That is a preference, I like being with my patients, I do not want to sit in front of a computer, that is not me, that is not medicine. [...] If it became mandatory, I would change jobs, I would not do this anymore*.

[Orthopedic Surgeon 5]

#### New Knowledge and Skills Are Needed to Use Software, Apps, and Devices

Using technology in care requires 2 key conditions: having the equipment and knowing how to use it. Digital literacy is a mandatory competency for both patients and health care providers in remote evaluations. Telehealth software specialists warned that technology could lead to errors, such as biased clinical measures or interpretations. All stakeholders, even patients, also warned that certain patient populations, such as older adults or individuals with less overall digital literacy, might have more difficulties with technology.

*Technology can play tricks on us. Because, for example, my camera has an automatic focus. If I look at a patient walking, then the patient goes out of focus, I have just lost the information I needed for my evaluation. Sometimes, the point of view I am going to get will not be the right one either. When training providers to use technology, they need to be aware of the limitations, so that they can address them in their evaluations and put in place adaptations to mitigate them*.

[Telehealth software specialist 1]

New challenges related to medical data confidentiality were also anticipated by all stakeholders.

#### Using Technology Means Dealing With and Solving Technical Problems

Health care providers, especially those who frequently use telehealth, pointed out the impact of technical problems during remote consultations, such as difficulties connecting or unstable internet connections. They highlighted the impact on the perceived quality of the consultation when technical problems happen and presented this type of problem as a reason why providers could be reluctant to use telehealth.

*Technical problems do not occur at every consultation, but it is really annoying when they do happen. It is a real waste of time for me and for the patient*.

[Physiotherapist 3]

Preparation was again reported as a strategy to mitigate the technical problems, such as sending connection links in advance. Telehealth software specialists emphasized the need for developing telehealth solutions and procedures that maximize usability, with only a few simple steps needed to establish a connection.

### Theme 4: Integrating Remote Evaluation Into the Health Care System: Opportunities and Challenges

The last theme highlights how integrating remote initial evaluations presents solutions to several challenges faced in health care systems, such as reducing pressure on health care services systems and addressing service gaps in underserved regions. However, administrative and organizational barriers could impede the successful widespread implementation.

#### Fully Remote Care Is an Opportunity to Alleviate System Pressure and to Improve Access to Health Care

Implementing remote evaluations is widely viewed as a solution to enhance health care coverage, particularly in remote areas where access to care is limited, especially for specialized medical services. However, remote evaluations may also offer benefits in urban areas, where health care providers are more readily available, by facilitating access to patients with limited mobility or transportation challenges.

*Over in XXX, it is a very, very large territory, so people can not always travel. Transportation is also very difficult, it is often by plane, by boat, basically any means except by car. So, there is no direct access to certain healthcare services. Obviously, in remote regions like that, telerehabilitation has always had its place*.

[Physiotherapy technologist 2]

Participants highlighted several advantages of telehealth in the management of MSKDs. It can reduce the need for unnecessary in-person consultations in first-line services, such as emergency departments, offer rapid remote triage to better address patient care needs and manage waiting lists, particularly for specialized services such as orthopedics, and increase overall system efficiency by minimizing travel times and costs, as well as reducing wait times in clinics. These improvements contribute to a more streamlined and accessible health care system.

*For people like me who work, it is important to avoid travelling. I have to stop work in the middle of the day, then take several public transport services. Doing it by videoconference would take less time. You have to go there in-person, you have to wait, it can take an hour, two hours. Whereas if it is by videoconference, the consultation sometimes takes 15 minutes, so the physician calls me when he is ready, we talk for fifteen minutes and then I do not lose two hours. It is a huge time-saver*.

[Patient 5]

#### Administrative and Organizational Barriers Within the Health Care System Still Need to Be Addressed

Some health care providers highlight that their organization itself can be a barrier to offering telehealth services. Many lack facilities suitable for telehealth or the basic equipment, such as webcams. Public insurance schemes could pose an important barrier to remote evaluations. They may require precise measurements such as range of motion and strength during assessments—measures which may be difficult to obtain remotely—or may not reimburse remote interventions, as reported by a representative of a professional organization.

[Quebec public insurance for work-related MSKDs] *requires us to have numbers, objective measurements. They ask me for written reports, but in these reports, I need a grip strength, I need a measure of range of motion. It is hard to come up with precise measures with the* [remote] *evaluation and when they ask me for my report the next time, I will not have any precise measures to give them and from there well the report cannot go through.*

[Physiotherapist 3]

Both patients and providers acknowledged the risk of duplication, where a remote evaluation might need to be followed by an in-person visit. Patients also raised practical concerns regarding the organization of care, such as how prescriptions would be sent or received, or how remote evaluation would be booked.

## Discussion

### Principal Findings

This qualitative study explored the perceptions of various stakeholders on the use of telehealth for the initial evaluation of adults with MSKDs, with a particular focus on conducting physical examinations remotely. Four main themes were identified aligned with the 4 framings described by Mathieu-Fritz and Esterle [29]: (1) a remote evaluation is often feasible by adapting the clinical examination (clinical framing), (2) a different patient-provider care relationship (social framing), (3) using information and communication technology efficiently (technical framing), and (4) integrating remote evaluations into the health care system: opportunities and challenges (organizational framing). Overall, all stakeholders regarded initial remote evaluation as an innovative, efficient, and generally safe approach to delivering MSKDs care for simpler cases, particularly for improving access to care for several underserved populations. However, they also acknowledged several limitations.

Remote physical examinations raised concerns among all stakeholders, particularly regarding the necessary adaptations and overall feasibility of conducting them remotely. Technology was also perceived as making evaluation more complex, with the potential to lead to errors. These concerns about the quality and safety of remote interventions have already been reported in the literature in relation to the loss of physical contact during remote care and the effectiveness of remote interventions for patients with MSKDs [21-23,25]. One of our findings was that the loss of physical contact, which could impact the diagnostic validity, was perceived by all stakeholders as having a greater impact on the initial evaluation than on subsequent remote follow-up, and it was perceived as the most important barrier to a remote evaluation. Both patients and health care providers expressed their intentions to use remote evaluations for what they consider simpler cases or patients with urgent needs. This perspective aligns with emerging evidence that suggests that remote diagnoses have been valid for lower or upper limb MSKDs and, in some cases, for more acute MSKDs [18].

The “digital interface” was perceived as negatively impacting the patient-provider care relationship. The therapeutic alliance was perceived by all as potentially weaker and more challenging to establish remotely due to barriers in communication and the loss of nonverbal cues. Both patients and health care providers expressed the importance of establishing adequate “webside manner” as a potential solution to better interact and successfully perform initial evaluations [26-28]. These difficulties have also been highlighted in the literature by others for remote interventions and follow-ups of patients with MSKDs [20,26,27,39]. However, a notable aspect of our results is that the initial remote evaluation appears to be more impacted by these relational difficulties, as it is the first encounter between patients and providers [24]. Again, even though both patients and health care providers were open to using remote evaluations for situations they considered simple or when patients had rapid access to care.

Our results highlight common concerns related to the use of technology, such as possessing the hardware and a basic level of digital literacy, technical problems, or data confidentiality [20,39,40]. Our findings also illustrate the new and potentially more demanding tasks inherent to the use of technology beyond the care intervention itself. This “invisible work” for the health care providers has already been described in the literature, but here it is in the context of the initial remote evaluation [41]. This heavier workload should be anticipated when planning to implement remote evaluation interventions and overall care. However, the added workload may be partially offset by the elimination of some tasks specific to in-person care such as welcoming and managing patients in waiting rooms or travel time for home visits by health care providers.

All stakeholders also reported the benefits of integrating remote evaluations in MSKD care pathways. Participants highlighted advantages such as reducing the health care system congestion, enhancing geographical service coverage, and better access for underprivileged populations. However, several organizational difficulties are identified, including how reimbursements would be processed for public insurance plans, a lack of organizational support, staff shortages, and limitations in space and equipment. While these benefits and organizational barriers have been documented in the literature for remote interventions [20,24,39,42], our study reveals organizational barriers that are specific to the initial evaluation, which are mainly related by regulations and requirements imposed by insurers (public, semipublic, or private), such as the obligation of objective evaluation measures that cannot be performed remotely. Advocacy efforts should be directed toward administrators and policy makers to overcome these administrative and legislative barriers.

Toolkits are available to support remote interventions beyond the initial evaluation [43,44], but our results emphasize the need to address specific gaps to promote the implementation and adoption of remote evaluations, the appropriate use of technology, and the evidence on the validity, safety, and limitations of remote evaluations. “Webside manners” should be included in these resources to foster effective collaboration during the remote physical examination and a strong therapeutic relationship between patients and providers [26-28].

### Strengths and Limitations

This study has several strengths. We followed the COREQ reporting checklist to ensure the transparency and reproducibility of the methodology [31]. We conducted several interviews with a wide range of stakeholders and profiles to ensure we achieved a more in-depth understanding of remote initial evaluations. We used 3 coders with diverse backgrounds, all experienced in qualitative analysis, to enhance the confirmability and the comprehension of the participant under study. Credibility and transferability of our results were enhanced by using 2 validated theoretical frameworks and by discussing coding trees and results in meetings with the principal researchers who have expertise in telehealth and MSKDs. However, there are few limitations to our study. Transcripts were not returned to participants for comment or correction, which may have affected the confirmability of our findings. However, our methodology involved the use of 2 conceptual frameworks, the involvement of 3 trained and experienced coders, and several team meetings to discuss and validate coding, analyses, and results. Not all physicians interviewed had prior experience with videoconferencing consultations, although most had used phone consultations. Among the patients, nearly half had no previous telehealth experience, but this provided the opportunity to explore their beliefs and concerns about telehealth evaluations, nonetheless. It would be relevant to explore, in the context of Quebec, the perceptions of non–French-speaking populations or other minorities, such as patients who speak only English, as they may have a different perspective on the benefits and challenges associated with the use of telehealth. Finally, we cannot exclude a social desirability bias that may have led participants to respond in favor of telehealth, but this was limited by the varied profile of the participants.

### Conclusions

Telehealth is perceived as a valuable solution to improve access to care for patients with MSKDs, particularly for simpler cases or patients with urgent needs. However, remote physical examination poses challenges related to its validity and feasibility due to the loss of patient-provider physical contact. Effective implementation will likely require more evidence-based guidelines, comprehensive provider training on remote techniques, and strategies to maintain patient-provider relationships. Addressing access to technology, digital literacy, and privacy concerns is also essential to ensure equitable adoption and optimize telehealth in musculoskeletal care. Future research should focus on developing and validating remote initial evaluation guidelines for various MSKDs and exploring the perceptions of patients and providers who have used remote evaluation to further develop fully acceptable and efficient remote care pathways.

## Supplementary material

10.2196/72901 Multimedia Appendix 1 Interview guides.

10.2196/72901 Checklist 1 COREQ checklist. COREQ: Consolidated Criteria for Reporting Qualitative Research.

## Abbreviations

COREQ: Consolidated Criteria for Reporting Qualitative Research
MSKD: musculoskeletal disorder
