# Supplementary material for: Telehealth for the Initial Evaluation of Musculoskeletal Disorders: Qualitative Study of Patients, Health Care Providers, and Key Stakeholders in the Province of Quebec in Canada
Source: J Med Internet Res. 2025 Jul 24;27:e72901. doi: 10.2196/72901 (PMC12288701; doi:10.2196/72901)
Supplement: Multimedia Appendix 1 [file jmir-v27-e72901-s001.docx]

1. Introductory word
2. Family physicians and MSK specialists
3. Physiotherapy professionals
4. Patients with MSKDs
5. Professional and regulation organizations
6. Telehealth software specialists

Hello,

I would like to thank you for agreeing to participate in this research project.

My name is ______ and I will be conducting your interview today.

**Project Objectives**

Before we begin the discussion, I would like to give you a brief reminder of the project you are participating in.

The general objective of this project is to study the possibility of conducting an initial remote evaluation, via videoconferencing, for patients with musculoskeletal disorders.

To ensure the intervention is appropriate for health care providers and patients, we are conducting semi-structured interviews with health care providers (*adapted to the type of participant*) who have either managed patients through telehealth or have never used telehealth, as well as interviews (*adapted to the type of participant*) with patients, professional organizations and telehealth software specialists.

This information will allow us to develop an evaluation method suitable for health care providers within the context of the Quebec health care system.

During this interview, we wish to explore your experiences and expectations related to the management of patients using telehealth including the initial evaluation.

I will ask you various questions about your possible experience and expectations related to telehealth. There are no right or wrong answers. I may clarify my question if needed.

Do you have any questions?

I will now start the recording.

.

| Question | Prompts |
| --- | --- |
| Could you briefly introduce yourself? | What is your professional background?  What is your practice environment?  Do you use telehealth? On which occasions? |
| **If experience with telehealth:**  Could you describe your experience with telehealth?  **If no experience:**  Could you describe your impressions on telehealth? | **If experience with telehealth:**  Which treatments did you offer/are you offering via telehealth? Does this correspond to what you offer in person? How did telehealth become part of your practice? Did the COVID-19 pandemic influence your relationship with telehealth?  Did you feel comfortable offering telehealth to your patients? Why? What level of confidence do you have in a telehealth treatment? What elements could impact this?  What was the biggest obstacle you encountered with telehealth? What strategy did you implement to overcome it? What other difficulties do you see? What did you do?  What changes did you have to make compared to your usual practice? Were you satisfied with the care you delivered? Why?  Would you imagine a completely remote management for musculoskeletal disorders? Why?  What do you think about remote follow-up for your patients with musculoskeletal disorders?  Do you think an initial remote evaluation is possible for musculoskeletal disorders? Why? What elements should this evaluation include? What elements would need to be adapted? How does this evaluation differ from other evaluations you might perform during treatment? Do you see any unique characteristics? Which ones?  Which body regions do you think are suitable for telehealth management? Why?  **If no experience:**    For what reasons do you not use telehealth?  Would you feel comfortable offering telehealth to your patients? Why?  What would be the biggest obstacle you see with telehealth? What strategy would you implement to overcome it? What other difficulties do you see? What would you do?  What changes would you need to make to your practice? Are they compatible with your practice? Why?  Would you imagine a completely remote management for musculoskeletal disorders? Why?  What do you think about remote follow-up for your patients with musculoskeletal disorders?  Do you think an initial remote evaluation is possible for musculoskeletal disorders? Why? What elements should this evaluation include? What elements would need to be adapted? How does this evaluation differ from other evaluations you might perform during treatment? Do you see any unique characteristics? Which ones?  Which body regions do you think are suitable for telehealth management? Why? |
| **If experience with telehealth:**  Could you describe the feelings of your patients?  **If no experience:**  In your opinion, what would be the feelings of your patients if you were to use telehealth? | **If experience with telehealth:**  Were they satisfied with your telehealth care? Why?  What level of confidence did you feel from your patients during telehealth sessions? What elements could impact this?  What difficulties did they encounter? What did they do to overcome them?  Has this modality of care changed your relationship with your clients/patients? Did the loss of physical contact have an impact? In what way?  **If no experience:**  Would they be satisfied with your telehealth care? Why?  What level of confidence would you feel from your patients during telehealth sessions? What elements could impact this?  What difficulties might they encounter? What could they do to overcome them?  Would this modality of care change your relationship with your clients/patients? Would the loss of physical contact have an impact? In what way? |
| Could you describe how your colleagues view telehealth? | Do your colleagues use telehealth? Why? Does this encourage you to use telehealth? Why?  Does your colleagues' opinion influence your practice? In what way?  Who else might influence your professional practice? |
| Could you describe the uses of telehealth by other health care providers that you have observed or can imagine? | What advantages can telehealth bring to other health care providers? And for their patients?  For which professions does the use of telehealth seem appropriate to you?  What are your thoughts on the care or evaluation of one of your patients via telehealth by another health care provider?  Does telehealth seem to you to be an appropriate tool for multidisciplinary care of patients? Why? |
| Could you describe your employer's or organization's stance on telehealth? | What is the role of telehealth in your organization?  How was telehealth introduced into your organization?  What are the added values of telehealth for your organization?  How would you have imagined the implementation of telehealth in your organization?  Are you encouraged to use telehealth? Are you required to use it? Why?  How do you see the future development of telehealth in your organization? |
| Could you tell me about the telehealth practice framework in your profession? | What are the modalities of practicing telehealth for you? Do you feel comfortable with them? Are there elements missing for you to integrate it into your professional practice? Which ones?  Do you feel encouraged to practice telehealth within your profession? Why? |
| How do you see the future of telehealth? | Are you satisfied with the possibilities offered by telehealth today? What elements are missing? What points need improvement?  How do you see the evolution of telehealth in the future? And in your own professional practice?  How do you see the inclusion of telehealth in the care pathway of your patients and the general population? |

| Question | Prompts |
| --- | --- |
| Could you briefly introduce yourself? | What is your professional background?  What is your practice environment?  Do you use telerehabilitation? On which occasions? |
| **If experience with telehealth:**  Could you describe your experience with telerehabilitation?  **If no experience:**  Could you describe your impressions of telerehabilitation? | **If experience with telerehabilitation:**  Which treatments did you offer/are you offering via telerehabilitation? Does this correspond to what you offer in person? How did telerehabilitation become part of your practice? Did the COVID-19 pandemic influence your relationship with telerehabilitation?  Did you feel comfortable offering telerehabilitation to your patients? Why? What level of confidence do you have in a telerehabilitation treatment? What elements could impact this?  What was the biggest obstacle you encountered with telerehabilitation? What strategy did you implement to overcome it? What other difficulties do you see? What did you do?  What changes did you have to make compared to your usual practice? Were you satisfied with the care you delivered? Why?  Would you imagine a completely remote management for musculoskeletal disorders? Why?  What do you think about remote follow-up for your patients with musculoskeletal disorders?  Do you think an initial remote evaluation is possible for musculoskeletal disorders? Why? What elements should this evaluation include? What elements would need to be adapted? How does this evaluation differ from other evaluations you might perform during treatment? Do you see any unique characteristics? Which ones?  Which body regions do you think are suitable for telerehabilitation management? Why?  **If no experience:**    For what reasons do you not use telerehabilitation?  Would you feel comfortable offering telerehabilitation to your patients? Why?  What would be the biggest obstacle you see with telerehabilitation? What strategy would you implement to overcome it? What other difficulties do you see? What would you do?  What changes would you need to make to your practice? Are they compatible with your practice? Why?  Would you imagine a completely remote management for musculoskeletal disorders? Why?  What do you think about remote follow-up for your patients with musculoskeletal disorders?  Do you think an initial remote evaluation is possible for musculoskeletal disorders? Why? What elements should this evaluation include? What elements would need to be adapted? How does this evaluation differ from other evaluations you might perform during treatment? Do you see any unique characteristics? Which ones?  Which body regions do you think are suitable for telerehabilitation management? Why? |
| **If experience with telerehabilitation:**  Could you describe the feelings of your patients?  **If no experience:**  In your opinion, what would be the feelings of your patients if you were to use telerehabilitation? | **If experience with telerehabilitation:**  Were they satisfied with your telerehabilitation care? Why?  What level of confidence did you feel from your patients during telerehabilitation sessions? What elements could impact this?  What difficulties did they encounter? What did they do to overcome them?  Has this modality of care changed your relationship with your clients/patients? Did the loss of physical contact have an impact? In what way?  **If no experience:**  Would they be satisfied with your telerehabilitation care? Why?  What level of confidence would you feel from your patients during telerehabilitation sessions? What elements could impact this?  What difficulties might they encounter? What could they do to overcome them?  Would this modality of care change your relationship with your clients/patients? Would the loss of physical contact have an impact? In what way? |
| Could you describe how your colleagues view telerehabilitation? | Do your colleagues use telerehabilitation? Why? Does this encourage you to use telerehabilitation? Why?  Does your colleagues' opinion influence your practice? In what way?  Who else might influence your professional practice? |
| Could you describe the uses of telehealth by other health care providers that you have observed or can imagine? | What advantages can telehealth bring to other health care providers? And for their patients?  For which professions does the use of telehealth seem appropriate to you?  What are your thoughts on the care or evaluation of one of your patients via telehealth by another health care provider?  Does telehealth seem to you to be an appropriate tool for multidisciplinary care of patients? Why? |
| Could you describe your employer's or organization's stance on telerehabilitation? | What is the role of telerehabilitation in your organization?  How was telerehabilitation introduced into your organization?  What are the added values of telerehabilitation for your organization?  How would you have imagined the implementation of telerehabilitation in your organization?  Are you encouraged to use telerehabilitation? Are you required to use it? Why?  How do you see the future development of telerehabilitation in your organization? |
| Could you tell me about the telehealth practice framework in your profession? | What are the modalities of practicing telerehabilitation for you? Do you feel comfortable with them? Are there elements missing for you to integrate it into your professional practice? Which ones?  Do you feel encouraged to practice telerehabilitation within your profession? Why? |
| How do you see the future of telerehabilitation? | Are you satisfied with the possibilities offered by telerehabilitation today? What elements are missing? What points need improvement?  How do you see the evolution of telerehabilitation in the future? And in your own professional practice?  How do you see the inclusion of telerehabilitation in the care pathway of your patients and the general population? |

| Question | Prompts |
| --- | --- |
| **If experience with telehealth:**  Could you tell me about your experience and share your impressions of telehealth?  **If no experience with telehealth:**  Could you describe your impressions of telehealth? | What advantages do you see in telehealth?  What was the biggest obstacle you encountered/would see with telehealth? How did you overcome it/would you overcome it? Do you see any others? Which ones?  Did you/would you feel comfortable receiving care remotely? Why?  What level of confidence do you have in remote care? What elements could impact this?  What changes do you see with in-person care? Is it bothersome for you? Why? How was your relationship with your health care provider? |
| What do you think about an initial remote evaluation for a musculoskeletal disorder? | Do you think an initial remote evaluation is possible for musculoskeletal disorders? Why? For which body regions or conditions? How do you imagine this evaluation? What limitations do you see in this evaluation?  How would you imagine this evaluation?  Would you imagine a completely remote management for a musculoskeletal disorder? Why? What do you think about remote follow-up for your care? |
| **If experience with telehealth:**  Could you describe how telehealth was introduced in your care?  **If no experience with telehealth:**  Could you describe how telehealth should be introduced in your care? | Who initiated/should initiate the use of telehealth in your care? How did you experience this?  OR  How would you have imagined/would you imagine the introduction of telehealth in your care?  Did you feel encouraged to use telehealth? In what way?  OR  Would you like to be encouraged and supported to use telehealth? By whom? |
| How do you imagine telehealth in the future? | Are you satisfied with the possibilities offered by telehealth today? What elements are missing? What points need improvement?  How do you see the evolution of telehealth in the future? And for you as a patient? |

| Question | Prompts |
| --- | --- |
| Could you briefly introduce yourself? |  |
| For you, what are the major challenges of this period? | What are the current needs of the population?  What are the expectations of the population?  Are there aspects of the healthcare system that need improvement? |
| What challenges does telehealth address, in your opinion? | What do you think about telehealth?  What advantages does telehealth bring, in your opinion?  What would be the biggest obstacle you see for telehealth? What strategy could be implemented to overcome it? What other difficulties do you see? What strategies should be adopted?  What limitations do you see in telehealth?  Would you imagine a completely remote management for musculoskeletal disorders? Why? What would be the conditions for this management?  What do you think about remote follow-up for this type of management? What would be the conditions for this follow-up?  Do you think an initial remote evaluation is possible for musculoskeletal disorders? Why? What elements should this evaluation include? What elements should be adapted? What would be the conditions for this evaluation?  Which body regions do you think are suitable for remote care? Why? |
| What are the uses of telehealth by health care providers? | Have health care providers embraced telehealth? Why?  For which health care professions do you think telehealth is appropriate? For all patients? For all situations?  Does the current framework seem conducive to health care providers adopting telehealth? Why? |
| What is your organization's stance on telehealth? | What roles does your organization have in relation to telehealth?  What impact does it have on the development of telehealth?  What feedback have you received from the field regarding telehealth? |
| What future do you see for telehealth? | How do you see the evolution of telehealth in the future?  Are there any changes that need to be made in the way telehealth has been implemented so far? If so, which ones?  How do you see the inclusion of telehealth in the care pathway of the general population? |

| Question | Prompts |
| --- | --- |
| Could you briefly introduce yourself? |  |
| For you, what are the major challenges associated with telehealth? | What needs does telehealth address?  What are the advantages of telehealth?  What are the limitations of telehealth?  What would be the biggest obstacle you see for telehealth? What strategy could be implemented to overcome it?  What other difficulties do you see? What strategies should be adopted? |
| What are the uses of telehealth by health care providers? | Have health care providers embraced telehealth? Why? What would need to be done for them to adopt telehealth more widely?  Does the current framework seem favorable for health care providers to adopt telehealth? Why?  Are there any changes that need to be made in the way telehealth has been implemented so far? If so, which ones?  For which health care providers, do you think telehealth is appropriate? For all patients?  What types of measures are currently feasible in telehealth? What adaptations can be made? What measures do you think are not feasible?  Would you imagine a completely remote management for musculoskeletal disorders? Why?  What do you think about remote follow-up for this type of management?  Do you think an initial remote evaluation is possible for musculoskeletal disorders? Why? What elements should this evaluation include? What elements should be adapted?  Which body regions do you think are suitable for remote care? Why? |
| What future do you see for telehealth? | How do you see the evolution of telehealth in the future? What technological advancements can we expect?  In your opinion, will new types of data be collectible in the future?  How do you see the inclusion of telehealth in the care pathway of the general population? |
