# Supplementary material for: Telehealth for the Initial Evaluation of Musculoskeletal Disorders: Qualitative Study of Patients, Health Care Providers, and Key Stakeholders in the Province of Quebec in Canada
Source: J Med Internet Res. 2025 Jul 24;27:e72901. doi: 10.2196/72901 (PMC12288701; doi:10.2196/72901)
Supplement: Checklist 1 [file jmir-v27-e72901-s002.pdf]

Consolidated criteria for reporting qualitative studies (COREQ): 32-item checklist

Developed from:

Tong A, Sainsbury P, Craig J. Consolidated criteria for reporting qualitative research (COREQ): a 32-item checklist for interviews and focus groups. International Journal for Quality in Health Care. 2007. Volume 19, Number 6: pp. 349 – 357

Item No	Guide Questions/Description	Reported on Page #
Domain 1: Research team and reflexivity		
Personal Characteristics		
1. Interviewer/ facilitator	Which author/s conducted the interview or focus group?	Data collection section
2. Credentials	What were the researcher's credentials? E.g., PhD, MD	Metadata
3. Occupation	What was their occupation at the time of the study?	Metadata
4. Gender	Was the researcher male or female?	Metadata
5. Experience and training	What experience or training did the researcher have?	Data analysis section
Relationship with participants		
6. Relationship established	Was a relationship established prior to study commencement?	Participants section
7. Participant knowledge of the interviewer	What did the participants know about the researcher? e.g. personal goals, reasons for doing the research?	Interview guides
8. Interviewer characteristics	What characteristics were reported about the interviewer/facilitator? e.g. Bias, assumptions, reasons and interests in the research topic	Data analysis section
Domain 2: study design		
Theoretical framework		
9. Methodological orientation and Theory	What methodological orientation was stated to underpin the study? e.g. grounded theory, discourse analysis, ethnography, phenomenology, content analysis	Data collection section
Participant selection		

Item No	Guide Questions/Description	Reported on Page #
10. Sampling	How were participants selected? e.g., purposive, convenience, consecutive, snowball	Participants section
11. Method of approach	How were participants approached? e.g., face-to-face, telephone, mail, email	Participants section
12. Sample size	How many participants were in the study?	Results section
13. Non-participation Setting	How many people refused to participate or dropped out? Reasons?	NA
14. Setting of data collection	Where was the data collected? e.g., home, clinic, workplace	Participants section
15. Presence of nonparticipants	Was anyone else present besides the participants and researchers?	NA
16. Description of sample	What are the important characteristics of the sample? e.g. demographic data, date	Tables 1 & 2
Data collection		
17. Interview guide	Were questions, prompts, and guides provided by the authors? Was it pilot tested?	Appendix 2
18. Repeat interviews	Were repeat interviews carried out? If yes, how many?	Data collection section
19. Audio/visual recording	Did the research use audio or visual recording to collect the data?	Data collection section
20. Field notes	Were field notes made during and/or after the interview or focus group?	Data collection section
21. Duration	What was the duration of the interviews or focus group?	Results section
22. Data saturation	Was data saturation discussed?	Data collection section
23. Transcripts returned	Were transcripts returned to participants for comment and/or correction?	NA
Domain 3: analysis and findings		
Data analysis		
24. Number of data coders	How many data coders coded the data?	Data analysis section
25. Description of the coding tree	Did the authors provide a description of the coding tree?	Figure 1
26. Derivation of themes	Were themes identified in advance or derived from the data?	Data analysis section
27. Software	What software, if applicable, was used to manage the data?	Data analysis section

Item No	Guide Questions/Description	Reported on Page #
28. Participant checking	Did participants provide feedback on the findings?	NA
Reporting		
29. Quotations presented	Were participant quotations presented to illustrate the themes/findings? Was each quotation identified? e.g., participant number	Results section
30. Data and findings consistent	Was there consistency between the data presented and the findings?	Results section
31. Clarity of major themes	Were major themes clearly presented in the findings?	Results section
32. Clarity of minor themes	Is there a description of diverse cases or a discussion of minor themes?	Results section
